# Invasive Trichoderma longibrachiatum breakthrough infection in a hematology patient

**DOI:** 10.1016/j.mmcr.2025.100709

**Published:** 2025-06-14

**Authors:** Yuri Vanbiervliet, Robina Aerts, Ellen Boon, Toine Mercier, Ann-Sophie Jacob, Marijke Peetermans, Koen Debackere, Katrien Lagrou, Johan Maertens

**Affiliations:** a Department of Haematology, University Hospitals Leuven, Leuven, Belgium; b Department of Microbiology, Immunology and Transplantation, KU Leuven, Leuven, Belgium; c University of Antwerp, Antwerp, Belgium; d Department of Haematology, AZ Sint-Maarten, Mechelen, Belgium; e Department of Laboratory Medicine and National Reference Center for Mycosis, University Hospitals Leuven, Leuven, Belgium; f Medical Intensive Care Unit, Department of General Internal Medicine, University Hospitals Leuven, Leuven, Belgium; g Laboratory of Experimental Hematology, Leuven, Belgium

**Keywords:** *Trichoderma longibrachiatum*, *Aspergillus fumigatus*, Fungal co-infection, Invasive mould infections, Olorofim, Hematology

## Abstract

*Trichoderma* species are emerging as pathogens, causing invasive fungal infections, particularly in immunocompromised individuals. We report the case of a 61-year-old neutropenic female with hepatosplenic T-cell lymphoma and profound neutropenia, who developed a breakthrough infection with *Trichoderma longibrachiatum* while receiving liposomal amphotericin B for probable invasive pulmonary aspergillosis. Despite combination antifungal therapy the patient ultimately succumbed to multiple organ failure. *Trichoderma longibrachiatum* and *Aspergillus fumigatus* were identified as causative fungal pathogens. Antifungal susceptibility testing of the *T. longibrachiatum* isolate revealed resistance to isavuconazole but susceptibility to amphotericin B, voriconazole, itraconazole and olorofim.

## 1 Introduction

*Trichoderma* species are filamentous fungi that are common plant saprophytes as well as dominant members of the soil-borne fungal community [1]. Previously mostly seen as contaminants, they have recently emerged as pathogens of invasive mould disease mostly in immunocompromised hosts, such as those with haematological malignancies and allogeneic hematopoietic cell transplant recipients (in whom crude mortality rates up to 80 % have been reported) [2,3]. In vitro, *Trichoderma* spp. exhibit rapid growth with a distinctive macroscopic appearance: initially cream-colored and quickly turning green due to abundant sporulation. Histologically these fungi present as colorless hyphae in affected tissues, similar to other pathogens causing hyalohyphomycosis such as *Aspergillus*, *Fusarium*, *Scedosporium*, *Purpereocillium*, *Acremonium*, *Penicillium*, and *Scopulariopsis* [4]*.* Lungs are most commonly affected organs (42 %), followed by the peritoneum (22 %) and the central nervous system (16 %). In invasive disease, *Trichoderma longibrachiatum* is the most frequently reported species, followed by *Trichoderma atroviride*, *Trichoderma bissettii*, *Trichoderma citrinoviride*, *Trichoderma harzianum*, *Trichoderma koningii*, *Trichoderma pseudokoningii* and *Trichoderma viride* [2].

## 2 Case presentation

A 61-year-old Caucasian female presented to the emergency department with fever, chills, productive cough with purulent sputum and a pancytopenia upon lab examination. Empiric broad-spectrum antibiotics were initiated and the patient was hospitalized at the hematology unit (day 0). Initial broad infectious serology testing for *hepatitis (A, B and C), Cytomegalovirus, Epstein-Barr virus, Hantavirus, dengue virus, human immunodeficiency virus, Mycoplasma pneumoniae, Borrelia, Treponema pallidum, Bartonella* and *Leptospira* was negative. Urine culture was negative, and multiple blood cultures revealed gram-positive bacteria (*Staphylococcus hominis* and S. *epidermidis*), leading to a more narrow targeted antibiotic therapy. Whole body positron emission tomography/computed tomography (PET/CT) revealed supra- and infra-diaphragmatic hypermetabolic lymphadenopathy and a hypermetabolic splenomegaly. A trephine biopsy demonstrated features of hemophagocytic lymphohistiocytosis (HLH), which prompted the initiation of high-dose corticosteroids. Subsequently, transjugular liver biopsy confirmed a diagnosis of hepatosplenic T-cell lymphoma of α/β T cells, for which chemotherapy with doxorubicin, cyclophosphamide and etoposide was initiated.

**Fig. 1:**
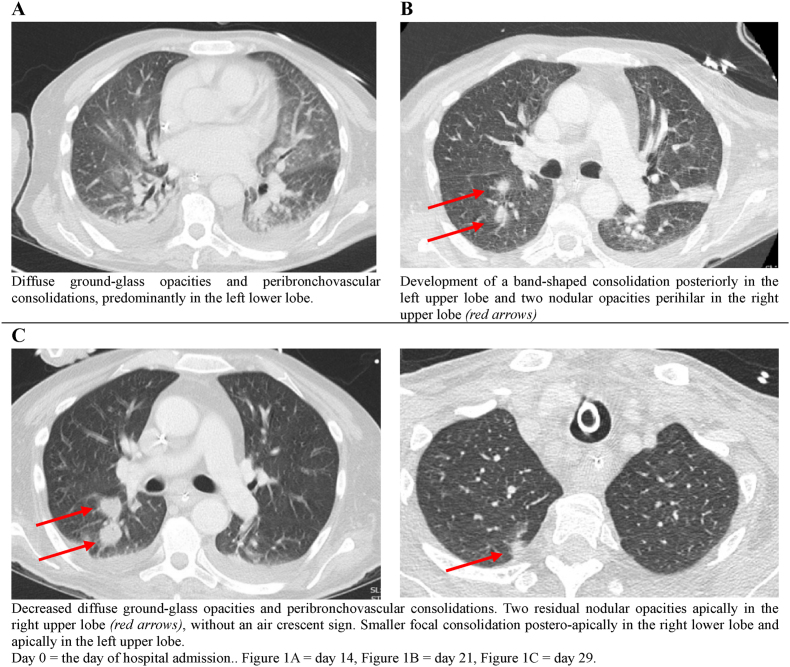
Radiological signs on chest CT.

Seven days after admission, the patient developed respiratory failure requiring intensive care unit (ICU) admission and mechanical ventilation. Prolonged profound neutropenia secondary to the underlying disease and its treatment led to successive infectious complications (including *Staphylococcus haemolyticus* bacteremia, neutropenic enterocolitis and *Enterococcus faecium* bacteremia) that were successfully managed with appropriate antibiotic therapy. However, following resolution of these bacterial infections, unexplained neutropenic fever persisted and the patient’s clinical condition deteriorated. Given her recent travel to high-risk regions for endemic mycoses in the USA, liposomal amphotericin B (L-AmB 3 mg/kg) was started empirically but rapidly discontinued as there were no arguments for an invasive fungal infection. However, on day 12, diagnosis of probable invasive pulmonary aspergillosis (IPA) was made based on rising serum galactomannan indices (GM; 0.70 optical density index [ODI]), positive bronchoalveolar lavage fluid (BALF) GM detection (>4.3 ODI) and *Aspergillus fumigatus* detected via polymerase chain reaction (PCR; Ct-value 35.5) on broncho-alveolar lavage fluid (BALF). None of the common *Aspergillus* resistance mutations (TR34/L98H or TR46/Y121F/T289A) were identified. Chest CT scan revealed diffuse bilateral ground-glass opacities and consolidations (Fig. 1A).

Treatment with isavuconazole (IV 200mg q8h day 1 and 2 and subsequent 200mg daily maintenance) was initiated but switched to L-AmB (3mg/kg) on day 17 due to signs of clinically progressive disease, rising serum galactomannan, and subtherapeutic isavuconazole trough levels. GM in serum and BALF normalized (ODI <0.5) suggesting a good therapeutic response, although chest CT showed the development of two new nodular opacities (Fig. 1B).

After eight days of L-AmB therapy (day 25), *Aspergillus* antigen in BALF turned positive again (>4.3 ODI) and was confirmed by repeat bronchoalveolar lavage on day 29. Nevertheless, serum GM continued to be negative (ODI <0.5). *Aspergillus* PCR in BALF remained positive for *A. fumigatus* (Ct-value 27.6) with persistently negative genotypic resistance testing. Simultaneously, fungal cultures from four separate BALF samples grew *Trichoderma longibrachiatum* (Fig. 3) whereas repeated chest CT showed reduced intensity of ground-glass opacities, consolidations and nodular lesions (Fig. 1C). Mucorales PCR, performed once on plasma and once on BALF, yielded negative results. Beta-D-glucan (BDG) on serum was only slightly positive (maximum value of 13.5pg/mL; normal value < 7pg/mL)).

**Fig. 2:**
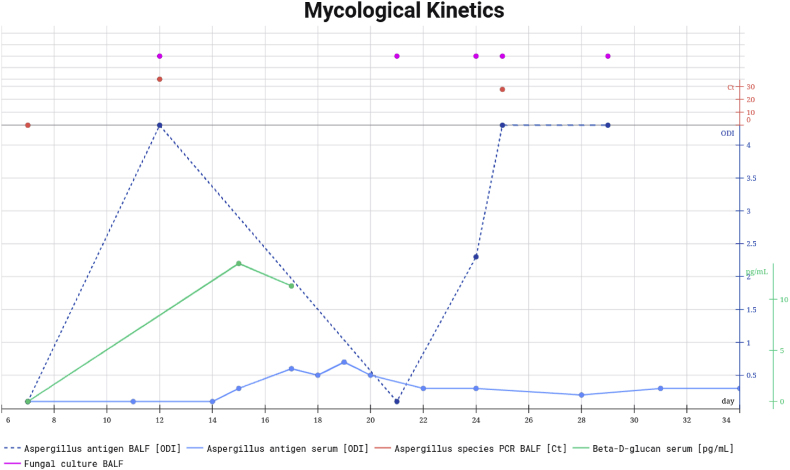
Mycological kinetics. All fungal cultures on BALF positive for fungi. Last four cultures grew T. *longibrachiatum*. *Aspergillus* species has not been cultured. Zero value for Aspergillus PCR and beta-D-glucan means ‘negative’. D7 is day of admission to the Intensive Care Unit, day 34 is day of death. Figure created in https://marpledata.com.

**Fig. 3:**
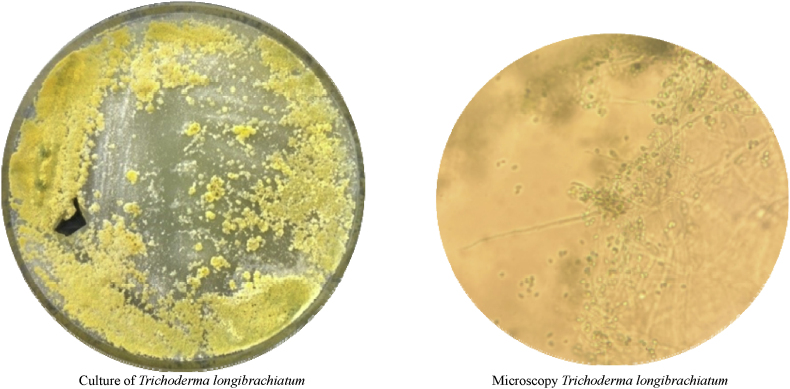
Macroscopy and microscopy of the isolate in casu.

Due to further clinical deterioration, the dosing of L-AmB was augmented to 5mg/kg and isavuconazole plus caspofungin (IV 70mg on first day, 50mg daily thereafter) were associated to the therapy. Despite these interventions, the patient's condition worsened further with the development of multiple organ failure and pneumatosis intestinalis. The patient eventually succumbed 34 days after admission to the hospital.

Radiological progression of the disease during ICU admission is detailed in Fig. 1.

Beta-D-Glucan (FUJIFILM Wako Chemicals Europe, Germany) was performed on serum. Galactomannan (GM)/*Aspergillus* antigen testing was performed serially on serum and all BALF samples using the Platelia *Aspergillus* assay (Bio-Rad Laboratories, Inc., USA). *Aspergillus* PCR testing on BALF was conducted with the AsperGenius assay (PathoNostics B.V., the Netherlands). This PCR detects *Aspergillus fumigatus*, *Aspergillus terreu*s, *Aspergillus flavus and Aspergillus* species and the major genetic mechanisms of azole resistance. Mucorales PCR on plasma and BALF was performed using the MucorGenius assay (PathoNostics B.V., the Netherlands). This PCR detects *Rhizopus* species, *Mucor* species, *Lichtheimia* species, *Cunninghamella* species and *Rhizomucor* species, accounting for 90 % of the species that cause invasive mucormycosis.

The kinetics of these mycological tests are summarized in Fig. 2. Macroscopy and microscopy of the *Trichoderma longibrachiatum* isolate are shown in Fig. 3.

Susceptibility of the *Trichoderma* isolate was tested using the broth microdilution method in RPMI medium, following the European Committee on Antimicrobial Susceptibility Testing (EUCAST) reference method. The isolate was tested for susceptibility to the following antifungal agents with their respective minimum inhibitory concentration (MIC) values: amphotericin B, 0.500 mg/L; isavuconazole, 4.00 mg/L; itraconazole, 1.00 mg/L; posaconazole, 0.500 mg/L; voriconazole, 0.500 mg/L; and olorofim, 0.016 mg/L. Susceptibility to echinocandins was not assessed. The MIC values for the tested antifungal agents are detailed in Table 1.

**Table 1:** Fungigram of Trichoderma longibrachiatum (isolated from BALF).

Antifungal	MIC-value (EUCAST [25])
**Amphotericin B**	0.500 mg/L
**Isavuconazole**	4.000 mg/L
**Itraconazole**	1.000 mg/L
**Posaconazole**	0.500 mg/L
**Voriconazole**	0.500 mg/L
**Olorofim**	0.016 mg/L

MIC = minimum inhibitory concentration.

## 3 Discussion

Invasive infections with *Trichoderma longibrachiatum* remain rare, but there has been an increase in reports of such infections over the last few decades [2,3,5]. Herein, we report an invasive co-infection with *A. fumigatus* and *T. longibrachiatum* in a patient with an underlying haematological malignancy, with detrimental outcome.

Fungal co-infections are common in immunocompromised patients [6]. In a prospective multicentric study, it was found that one-third (33.3 %) of mucormycosis cases were actually mixed *Aspergillus*-*Mucorales* infections [7]. A retrospective multicentric study showed a co-infection with *Aspergillus* in 50 % of patients with culture-positive probable and proven invasive mucormycosis [8]. Additionally, up to 10 % of invasive aspergillosis (IA) cases are associated with mucormycosis [9]. In this case, there was evidence of a co-infection with *Aspergillus fumigatus* and *Trichoderma longibrachiatum*. Based on the mycological kinetics (Fig. 2), it is plausible that the patient was initially infected with *Aspergillus* which was appropriately treated as indicated by the decline of GM in serum and BALF. Subsequently, the patient developed a breakthrough infection with *Trichoderma*. Although *Aspergillus* species were not isolated from cultures, PCR testing was positive twice for *A. fumigatus* in the BALF, and serial GM testing of serum and BALF samples remained positive, which aligns with a diagnosis of probable invasive aspergillosis according to the updated EORTC/MSGERC criteria [10]. After the normalization of GM, levels increased again in the BALF. *Trichoderma* species can potentially cause ‘false-positive’ *Aspergillus* antigen results in BALF specimens [11]. Furthermore, *Trichoderma* species may exhibit morphological features similar to those of hyalohyphomycosis on microscopy, which can lead to a misdiagnosis of aspergillosis [4]*.* The second rise in GM levels in the BALF may have been due to the co-infection with *Trichoderma*, or possibly refractory aspergillosis despite appropriate treatment, given the patient's severely immunocompromised state.

Susceptibility data for *Trichoderma* species remain scarce and demonstrate varying antifungal activity profiles [2,3,5]. In a scoping study, susceptibility values of 17 isolates of *T. longibrachiatum* were reported. The median (interquartile range [IQR]) minimum inhibitory concentration (MIC) values were as follows: amphotericin B (1.33 [1.0, 2.0] mg/L, *N* = 16), caspofungin (0.50 [0.205, 0.875] mg/L, *N* = 10), anidulafungin (0.12 [0.0675, 0.12] mg/L, *N* = 3), micafungin (0.06 [0.034, 0.06] mg/L, *N* = 3), fluconazole (64.0 [40.0, 72.0] mg/L, *N* = 7), posaconazole (5.0 [4.0, 7.5] mg/L, *N* = 6), voriconazole (0.5 [0.5, 1.0] mg/L, *N* = 13), isavuconazole (2.0 mg/L, *N* = 1), itraconazole (8.0 [1.25, 32.0] mg/L, *N* = 13) and flucytosine (41.0 [25.5, 101.5] mg/L, *N* = 6) [3]. Although methodologies for susceptibility testing differ between studies, clinical isolates of *T. longibrachiatum* generally exhibit resistance to flucytosine, fluconazole, and posaconazole, variable resistance to itraconazole, voriconazole, and amphotericin B and susceptibility to echinocandins [3,5].

Based on the clinical breakpoints for more virulent *Aspergillus* species [12], the MIC values for the current clinical isolate of *T. longibrachiatum* were interpreted as resistant to isavuconazole and posaconazole but susceptible to amphotericin B, voriconazole, and itraconazole, which is consistent with the aforementioned data. Olorofim, a novel first-in-class orotomide antifungal agent, has demonstrated promising activity against rare and difficult-to-treat molds [13]. Although clinical breakpoints for olorofim have not been established, its low MIC value (0.016 mg/L) in this study suggests susceptibility [14,15]. To the best of our knowledge, this is the first report of *in vitro* susceptibility of a clinical *T. longibrachiatum* isolate to olorofim.

In this case, the organs involved were the lungs and possibly the gastrointestinal tract, as evidenced by the development of extensive pneumatosis intestinalis shortly before the patient’s death. Lung involvement is the most common presentation of invasive *Trichoderma* infections (42 %), followed by peritoneal involvement (22 %) [2]. Risk factors in our case—hematological malignancy, corticosteroid use, neutropenia, and chemotherapy—are frequently observed in patients with *Trichoderma* infections [2].

Clinical outcomes in invasive *Trichoderma* infections are poor, with mortality rates reaching 80 % in allogeneic hematopoietic stem cell transplant recipients and 77 % in solid organ transplant recipients [2]. In a murine model using cyclophosphamide-immunosuppressed male OF-1 mice, *T. longibrachiatum* exhibited low virulence but significant clinical resistance to amphotericin B, voriconazole and micafungin, resulting in high mortality and poor therapeutic responses [16]. Mortality rates vary with different monotherapies: 53 % with lipid formulations of amphotericin B, 46 % with voriconazole, 80 % with itraconazole and 28 % with caspofungin. Mortality rates remain high despite combination therapies of azoles with amphotericin B, or voriconazole with caspofungin, with rates of 66 % and 25 %, respectively [2].

Due to the limited clinical data, no specific guidelines exist for treating invasive *Trichoderma* infections. Current international guidelines for rare mould infections recommend triazoles or lipid formulations of amphotericin B as first-line treatments [17,18]. Based on previous susceptibility data and our findings, empirical treatment with voriconazole, itraconazole or amphotericin B could be considered. However, itraconazole is less favorable due to its variable pharmacokinetics and tolerability [19]. The efficacy of isavuconazole remains unclear due to insufficient data. Echinocandins, with their favorable toxicity and drug interaction profiles, may be considered for combination therapy but should not be used as monotherapy due to their fungistatic nature against molds [20]. Further therapy should be guided by susceptibility data; however, it is difficult to correlate clinical response to antifungal drugs with their *in vitro* activity due to the limited number of cases with available information and the lack of clinical breakpoint values of antifungals for *Trichoderma* species.

Our patient was ultimately treated with the combination of liposomal amphotericin B, isavuconazole, and caspofungin. Susceptibility testing showed resistance to isavuconazole and susceptibility to amphotericin B, voriconazole, itraconazole, and olorofim. Considering susceptibility data and clinical characteristics, therapy with olorofim (administered orally or via nasogastric tube [21]) in combination with caspofungin may have been an attractive treatment option for this patient. Olorofim, in particular, would have been a suitable option for the treatment of both the *A. fumigatus* and *T. longibrachiatum* infection in this case, especially in patients with renal and hepatic dysfunction and risk of drug-drug interactions where amphotericin B [22] and triazoles are less favorable [23], but caution is needed with olorofim in the case of hepatic events [24].

## CRediT authorship contribution statement

**Yuri Vanbiervliet:** Conceptualization, Methodology, Writing – Original Draft, Visualization. **Katrien Lagrou:** Conceptualization, Writing – Review & Editing, Visualization. **Johan Maertens:** Conceptualization, Supervision, Writing – Review & Editing. **Ann-Sophie Jacob:** Visualization, Writing – Review & Editing. **Robina Aerts, Ellen Boon, Toine Mercier, Marijke Peetermans, Koen Debackere:** Writing – Review & Editing.

## Consent

Written informed consent was obtained from the patient or legal guardian(s) for publication of this case report and accompanying images. A copy of the written consent is available for review by the Editor-in-Chief of this journal on request.

## Funding source

NA.

## Conflict of interest

The authors declare that they have no conflicts of interest related to this manuscript.
